# Typhoid Fever

**Published:** 1859-10

**Authors:** 


### TYPHOID FEVER.

Space does not allow us to present to our readers the entire article or report of cases by Dr. AUSTIN FLINT, (as found in the New Orleans *Medical News & Hospital Gazette*); but, with this apology, we give below his "General Remarks," or summation of practical hints after the careful study of nine cases of Typhoid Fever, as presented in the New Orleans Charity Hospital. We give place to them the more cheerfully, perhaps, because in exact accordance with our own views and experience upon this subject:

"The nine cases of typhoid fever which have been reported, occurred in my wards during a service of three months. My wards contained about thirty beds. The means of determining the number of cases occurring during the same period in the other wards of the Hospital, are not at hand; but assuming an equal number to have occurred in the service of all the visiting physicians, the whole number of cases would exceed one hundred. The yearly report of the officers of the hospital to January, 1859, showed one hundred and twenty-nine cases of typhoid fever. This form of fever, thus, is sufficiently common in New Orleans. The proportion of cases to the number of patients received, is probably not far from the the average in Northern hospitals. The disease presents the same symptomatic phenomena as at the North. Judging from the histories of the cases now reported, there are no features peculiar to typhoid fever, as observed in New Orleans. It occurs here, as at the North, during the autumnal months. My service commenced in the middle of November, and of the nine reported cases five occurred during the latter half of this month; one case was admitted December 4th, and the remaining three cases in January. It is probable that of the 129 cases which were received during the year, the greater portion occurred during my term of service. It may be worthy of note that my service commenced just at the close of a yellow fever epidemic, which had been unusually severe and protracted, two thousand seven hundred and twenty-seven cases having been treated in the Charity Hospital. The fatality from typhoid fever, judging from these few cases, is not greater than in Northern cities. Of the nine cases, but one proved fatal, and in the only fatal case death was owing to an event which occurs in a certain proportion of cases, viz: apoplectic coma.

The indications for treatment are the same at New Orleans as elsewhere. The treatment pursued in the cases reported was simple and uniform, but certainly not devoid of efficiency. In all cases, save one,* it consisted in addition to opium or the sulphate of morphia administered by the mouth or rectum, in doses sufficient to keep the diarrhœa in check, of sustaining measures, in other words, of brandy and concentrated nourishment. These measures were pressed in proportion to the evidence afforded by the symptoms of the extent to which the vital powers were compromised by the disease. Brandy given, as in one of the cases, in doses of two ounces hourly, becomes a remedy of considerable potency. Given in much smaller quantity, it can hardly be considered inefficient by those (still not a few) who, regarding it as an incendiary remedy, withhold it entirely in the management of fevers and

*In the excepted case the sulphate of quinia was given at the commencement, the patient having quite recently had an attack of intermittent fever.

inflammations. With our present knowledge of the natural history and pathology of typhoid fever, together with the fruits of therapeutical experience, the indications for efficient treatment are fulfilled by sustaining measures graduated in each case by the state of the vital forces and the tendency to a fatal result by asthenia; and the sustaining measures which meet the indications most efficiently, consist of alcoholic stimulants in the form of wine or spirits, with certain forms of nourishment, embracing in concentrated bulk the important alimentary principles. The animal essences and milk furnish these principles best combined for nutrition. The sustaining plan of treatment carried out promptly, judiciously, and, if need be, boldly, together with palliative remedies suited to the circumstances in individual cases, will, I am satisfied, save not a few patients who would otherwise perish from typhoid fever; and when not essential to the safety of life, it conduces to a rapid and complete recovery.

*Remarks on One Case.*—An interesting point in the history of this case is referred to in the following note appended to the record: "This case illustrates the importance, in certain instances, of rousing patients to voluntary exertion. The patient lay day after day in bed, never speaking, and scarcely moving. He seemed almost inanimate, manifested no desire for food, and occasionally vomited. I directed the nurse to take him out of bed, and cause him to sit up for a little while daily. The first time he fainted. He was near fainting the second time, while I was in the ward. However, the plan was persevered in, and frictions made over the body. In a few days he manifested improvement, and has since convalesced rapidly."

---

### DISCOVERY OF THE ANÆSTHETIC EFFECTS OF CHLOROFORM.

A writer in the Household Words gives the following description of the discovery of the powerful anæsthetic properties of chloroform;

To Professor Simpson, of Edinburgh, belongs the distinguished credit of introducing chloroform, which has nearly superseded all other anæsthetics. Possessed with the notion that something better than ether existed in the chemieal world, the Professor set about deliberately to examine any volatile substance which afforded a promise of revealing the required properties. Various gases and liquids were experimented upon; and at last chloroform—a ponderous liquid which provoked no great expectations, and only known as a chemical curiosity in the laboratory—was brought to the trial. Dr. Simpson, with his two assistants, sat down late one night, after an arduous day's toil, and when most physicians, as well as patients, were wrapt in sleep, began to inhale various substances which had been collected. A small bottle of chloroform had been raked up out of some obscure corner, and was to take its turn with the rest. Each experimenter provided himself with a tumbler or finger-glass, a portion of each selected fluid was poured into the bottom of it, and the glass was placed over warm water, to favor evolution of vapor. Holding the mouth and nostrils over the vessel, these votaries of science courageously explored this *terra incognita* by inhaling one vapor after another. At last each one charged his tumbler from the small bottle of chloroform, "when immediately," says Professor Miller, "an unwonted hilarity seized the party; they became bright-eyed and very happy, and conversed with such intelligence as more than usually charmed other listeners who were not taking part in the proceedings. But suddenly there was a talk of sounds being heard like those of a cotton mill, louder and louder; a moment more, then all was quiet, and then—crash.

"On awaking, Dr. Simpson's first perception was mental: 'This is far stronger and better than ether,' he said to himself. His second was to note that he was prostrate on the floor, and that his friends were confused and alarmed. Hearing a noise, he turned round, and saw his assistant, Dr. Duncan, beneath a chair, his jaw dropped, his eyes staring, and his head half bent under him; quite unconscious, and snoring in a determined and alarming manner. More noise still, and much motion, and then his eyes overtook Dr. Keith's feet and legs making valorous efforts to overturn the table, or more probably to annihilate everything that was on it.

"All speedily regained their senses, and from that day, or rather from the middle of that night, dates the discovery of the marvellous properties of chloroform."—*Char. Med. Journal.*

